# AGING DISPLAYED IN SELF-PORTRAITS OF ARTISTS

**DOI:** 10.1093/geroni/igz038.642

**Published:** 2019-11-08

**Authors:** Manfred Gogol

**Affiliations:** Heidelberg University, Heidelberg, Germany

## Abstract

A self-portrait is a common representation of an artist with different techniques by that artist and emerged since the 15th century. Albrecht Dürer was one of the first artists who performed various self-portraits during his life. The fascinating aspect from a gerontological point of view is that artists show themselves throughout the aging process as well as sometimes with manifest signs of diseases. The poster show self-portraits over the life course from Rembrandt van Rijn (1606-69), Vincent van Gogh (1853-90), Ferdinand Hodler (1853-1918), Lovis Corinth (1858-1925), Helene Schjerfbeck (1862-1946), Edvard Munch (1863-1944), Käthe Kollwitz (1867-1945), Pablo Picasso (1881-1973), and Max Beckmann (1884-1950).

